# Long-term outcomes from the UK Biobank on the impact of coffee on cardiovascular disease, arrhythmias, and mortality: Does the future hold coffee prescriptions?

**DOI:** 10.21542/gcsp.2023.13

**Published:** 2023-05-11

**Authors:** Kotit Susy

**Affiliations:** Aswan Heart Centre (AHC), Aswan, Egypt

## Abstract

Introduction: Coffee is a popular beverage and the most used psychostimulant worldwide. Habitual coffee consumption has been linked to a growing list of health benefits; however, coffee consumption has been a source of longstanding debate. Recent observational studies have challenged the misconception of caffeine and reported the safety and beneficial effects of coffee intake on a range of cardiovascular (CV) conditions, including coronary artery disease, arrhythmias, heart failure, and stroke, leading to a decreased risk of CVD, all-cause and CVD mortality, and being associated with favorable CV outcomes. However, the mechanisms underlying the protective effects of caffeine remain speculative, and there is a lack of dedicated studies aimed at addressing the impact of different coffee subtypes on clinical outcomes such as CVD, arrhythmia, and mortality.

Study and Results: The study included 449,563 UK Biobank participants, free of cardiovascular problems at enrollment (median age 58 years; 55.3% females). The median follow-up time was 12.5 years. Drinking 4 to 5 cups/day of ground (HR 0.83; 95% CI [0.76–0.91];  *P*  < .0001) or 2 to 3 cups/day of instant (HR, 0.88; 95% CI [0.85–0.92];  *P*  < .0001) coffee (but not decaffeinated coffee) was associated with a significant reduction in incident arrhythmia, including AF. Habitual coffee intake of up to 5 cups/day was associated with significant reductions in the risk of incident CVD, CHD (HR 0.89, CI [0.86–0.91], *P* < 0.0001), CCF (HR 0.83, CI [0.79–0.87], *P* < 0.0001), and ischemic stroke (HR 0.84, CI [0.78–0.90], *P* < 0.0001). Coffee consumption led to significant reductions in all-cause mortality (HR 0.86, CI [0.83–0.89], *P* < 0.0001) and CV mortality (HR 0.82, CI [0.74–0.90], *P* < 0.0001). Consumption of ground coffee at all levels significantly reduced the risk of all-cause and CV mortality. There was no significant difference in the incidence of CVD among all intake categories or across all coffee subtypes.

Lessons learned: The results from the UK Biobank indicate that mild-to-moderate consumption of all types of coffee is linked to improved CV outcomes and a lower risk of cardiovascular disease and death, with caffeinated coffee significantly reducing the risk of arrhythmias, including AF. Daily coffee intake should not be discouraged by physicians, even in the presence of a newly developed cardiovascular disease. Whether coffee will be prescribed in the future to prevent CVD and improve cardiovascular health depends on further evaluation of the physiological mechanisms and elucidation of the specific protective effects of coffee consumption.

## Introduction

**Figure 1.**
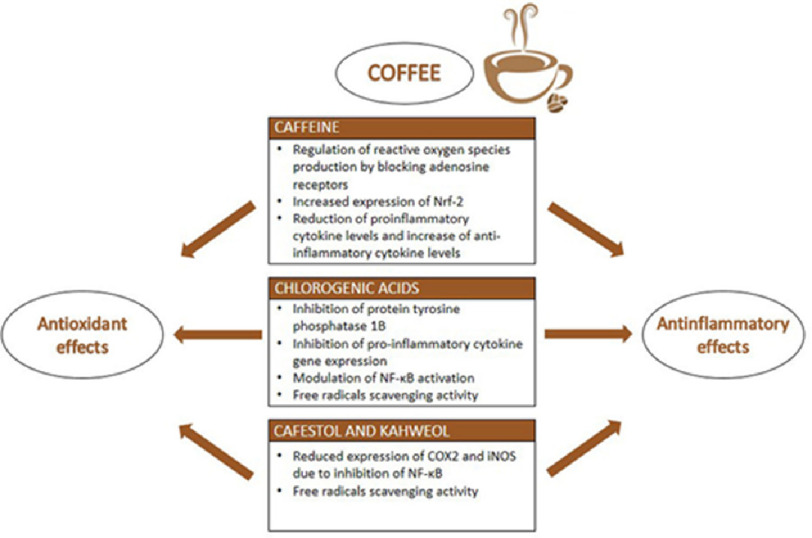
The main compounds of coffee: caffeine, chlorogenic acids, and other phenolics, and their anti-inflammatory and antioxidant effects^19^.

**Figure 2.**
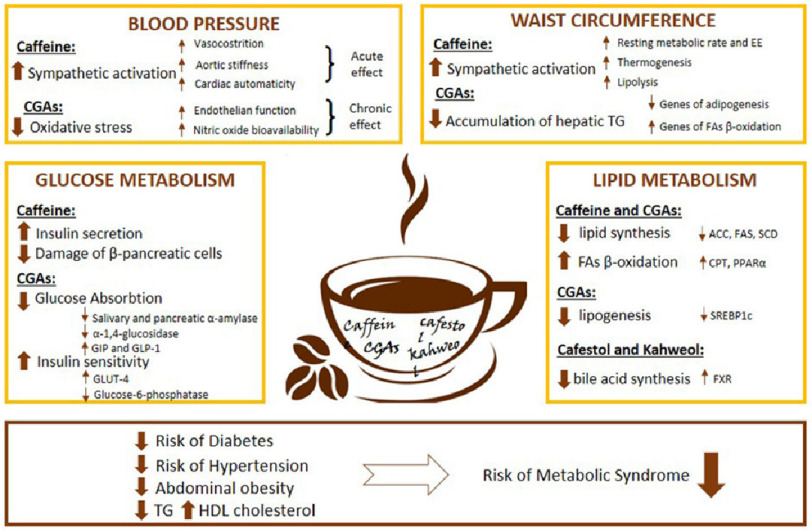
Summary of all major effects of coffee constituents on glucose and lipid metabolism, blood pressure, and waist circumference. Coffee contains many biologically active substances, including caffeine, chlorogenic acids (CGAs), and diterpenes as cafestol and kahweol, which exert different metabolic effects. Regarding glucose metabolism, caffeine effect insulin release, predominantly, increasing b-cells secretion and reducing their damage. At the same time, CGAs have different effects on glucose absorption and insulin sensitivity by inhibiting salivary and pancreatic a-amylase, a-1,4-glucosidase, and glucose-6-phosphatase secretion of incretins and by inducing the translocation of GLUT-4, responsible for glucose uptake by peripheral tissues. The acute hypertensive effect of coffee is mainly mediated by caffeine, stimulating sympathetic activation with vasoconstriction, increased aortic stiffness, and cardiac automatism, while in the long-term exposure, CGAs reduce oxidative stress by improving endothelial function and the bioavailability of nitric oxide, resulting in a reduction in blood pressure following chronic coffee intake. Regarding the effect of coffee consumption on waist circumference, caffeine stimulates the sympathetic nervous system increasing resting metabolic rate and energy expenditure, and promotes cellular thermogenesis and lipolysis. Simultaneously, CGAs act to suppress the accumulation of hepatic triglycerides *via* down-regulation of genes associated with adipogenesis and up-regulation of genes involved in fatty acid oxidation. Finally, about lipid metabolism, caffeine and CGAs have been shown to suppress the activity of enzymes for lipid synthesis, acetyl-CoA carboxylase, fatty acid synthase, and stearoyl-CoA desaturase, and to increase fatty acid b-oxidation by stimulating carnitine palmitoyltransferase and by activating PPAR-a in the liver and adipose tissues. Meanwhile, CGAs alone can control lipogenesis by down-regulating sterol regulatory element-binding protein (SREBP)-1C. Cafestol and kahweol have been shown to increase blood cholesterol levels by inhibiting bile acid synthesis acting on farnesoid X receptors. Therefore, habitual coffee consumption has been associated with a reduction in the risk of diabetes, hypertension, abdominal obesity, a reduction in triglycerides levels, and increased HDL cholesterol levels, resulting in a reduction in the risk of metabolic syndrome overall^19^.

Coffee is a popular beverage and the most commonly used psychostimulant worldwide^1^. In addition to being a delicious booster of energy levels, habitual coffee consumption has been linked to a growing list of health benefits, including the prevention of chronic and degenerative conditions, including cancer^2–4^; inflammatory and oxidative stress-related diseases such as obesity^5–7^, metabolic syndrome^7,8^, type 2 diabetes mellitus (DM)^9–17^, and autoimmune diseases including endocrine disease^18,19^, Parkinson’s^21–23^ and Alzheimer’s disease^24,25^ (Figures 1 and 2).

Coffee contains more than 100 different biological agents and many beneficial compounds for health. Caffeine, the most commonly studied compound in coffee, exerts positive effects on kidney function, and daily coffee consumption is associated with a lower risk of chronic kidney disease^26^ and lower risk of incident acute kidney injury (AKI), presenting an opportunity for cardiorenal protection through diet^27^. Although other compounds in coffee are less studied, compounds such as chlorogenic acid and trigonelline are known to reduce generalized inflammation and oxidative stress^28^.

Nevertheless, the health benefits and risks of coffee consumption have been a source of longstanding debate. Moreover, coffee and caffeine are often considered by the general population to be ‘bad’ for the heart owing to their association with palpitations and high blood pressure, and up to 80% of health practitioners recommend avoiding caffeine use in the presence of cardiovascular disease (CVD)^29^.

**Figure 3.**
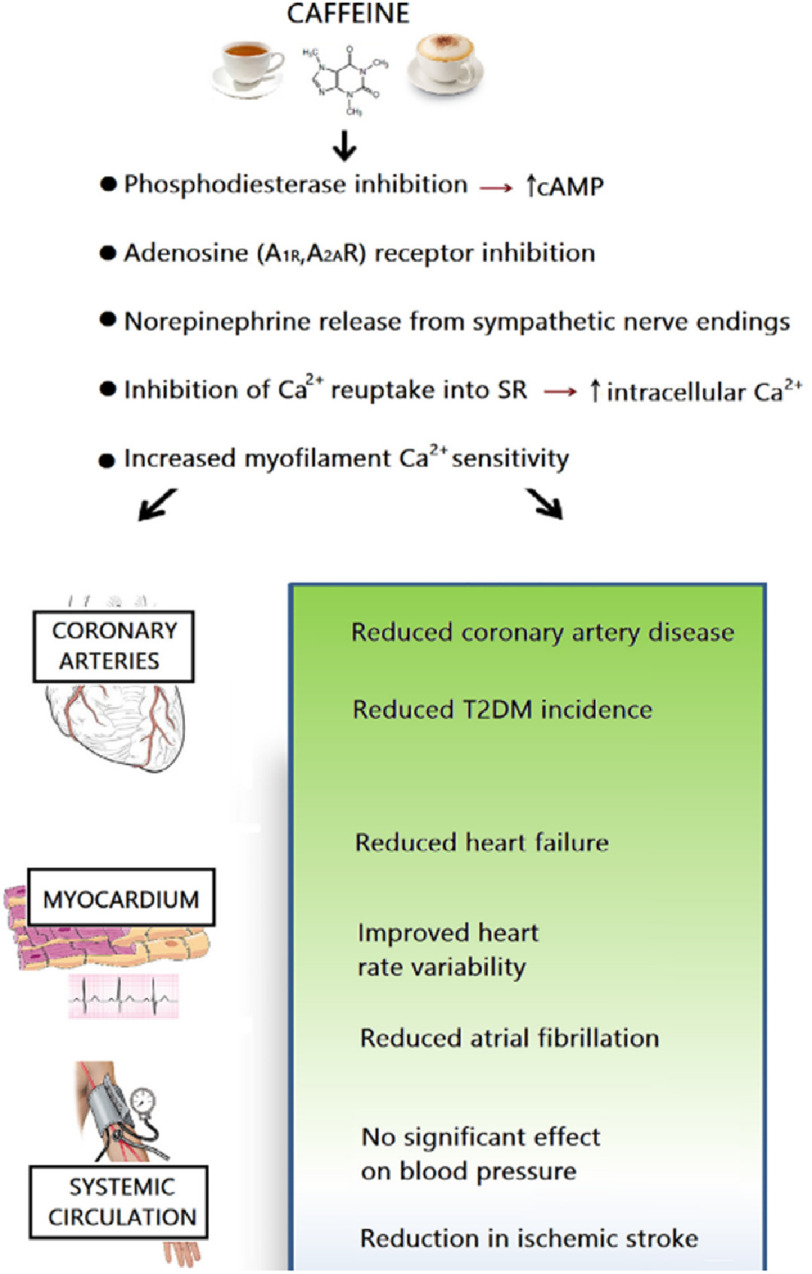
Physiological effects of habitual caffeine consumption on the cardiovascular system. Adapted from^33^.

Increasing public awareness of modifiable lifestyle risk factors has encouraged more information on the health benefits and CVD risks associated with coffee consumption. Recent observational studies have challenged the misconception of caffeine and reported the safety and beneficial effects of coffee intake^30–32^. Light-to-moderate coffee consumption(0.5–3 cups per day) has been shown to be beneficial for a range of cardiovascular conditions, including coronary artery disease(CHD)^33–35^, arrhythmias^33,36,37^ heart failure^20,33,38^ and stroke^30,32,37–41^ leading to a decreased risk of CVD^30–34,38,42,43^, all-cause^40,42,44–47^ and CVD mortality^48–51^ and being associated with favorable CVD outcomes (Figure 3).

**Figure 4.**
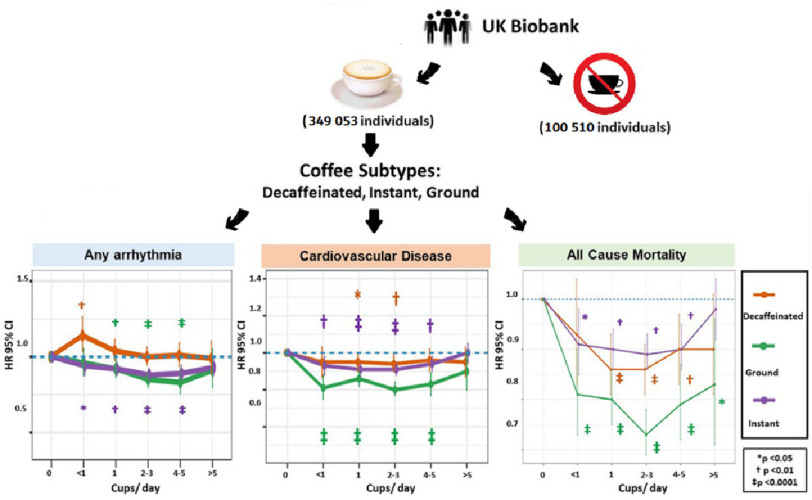
The impact of coffee subtypes on incident cardiovascular disease, arrhythmias, and mortality: long-term outcomes from the UK Biobank.

**Table 1:** Cardiovascular outcomes and risk reduction in different coffee subtypes and overall coffee intake.

Cardiovascular Outcomes	Ground Coffee		Instant Coffee		Decaffeinated Coffee		Overall Coffee Intake
	*n* = 82575	%	*n* = 198062	%	*n* = 68416	%	*n* = 349053
Arrhythmia	5872	7.0	16696	8.4	6737	9.8	
AF/flutter	3269	3.9	9273	4.7	3889	5.7	
CVD	8670	10.5	29751	15	9904	14.5	
CHD	7154	8.6	25051	12.6			
CCF	1976	2.3	7029	3.5	2263	3.3	
Stroke	1114	1.3	3707	1.8	1224	1.7	
Mortality	4511	5.5	15365	7.7	7434	10.9	
**Risk reduction effect**							
Arrhythmia	1–5 cups/day		2–3 cups/day 0.85–0.92 (HR 0.88, CI [0.85–0.92]				2–3 cups/day (HR 0.91, CI [0.88–0.94], *P* < 0.0001)
AF/flutter	1–5 cups/day		4–5 cups/day (HR 0.85, CI [0.79–0.91], *P* < 0.0001)				4–5 cups/day (HR 0.88, CI [0.83–0.94], *P* < 0.0001)
SVT	2–5 cups/day		4–5 cups/day (HR 0.75, CI [0.63–0.88], *P* = 0.0005)				
VT/VF	2–5 cups/day						4–5 cups/day (HR 0.83, CI [0.70–0.97], *P* = 0.0201)
CVD	up to 5 cups/day		2–3 cups/day (HR 0.91, CI [0.88–0.94], *P* < 0.0001)		2–3 cups/day (HR 0.94, CI [0.90–0.99], *P* = 0.0093)		5 cups/day
CHD	up to 5 cups/day		2–3 cups/day (HR 0.91, CI [0.88–0.94], *P* < 0.0001)		2–3 cups/day (HR 0.94, CI [0.89–0.99], *P* = 0.0127)		2–3 cups/day (HR 0.89, CI [0.86–0.91], *P* < 0.0001).
CCF	up to 5 cups/day				2–3 cups/day (HR 0.86, CI [0.79–0.94], *P* = 0.0004)		2–3 cups/day (HR 0.83, (CI [0.79–0.87], *P* < 0.0001)
All-cause mortality	2–3 cups/day (HR 0.73, CI [0.69–0.78], *P* < 0.0001)		2–3 cups/day (HR 0.89, CI [0.86–0.93], *P* < 0.0001)		2–3 cups/day (HR 0.86, CI [0.80–0.91], *P* < 0.0001)		2–3 cups/day (HR 0.86, CI [0.83–0.89], *P* < 0.0001)
CV mortality	4–5 cups/day (HR 0.65, CI [0.51–0.83], *P* < 0.0001)				1–3 cups/day (HR 0.74, CI [0.61–0.89], *P* = 0.0012)		1 cup/day (HR 0.82, CI [0.74–0.90], P 0.0001)

Importantly, caffeine consumption has been shown to be unlikely related to the incidence or the risk of developing atrial fibrillation^52,53^, even possibly offering a protective effect^54^. In addition, moderate and habitual consumption of coffee (1–3 cups per day) has recently been shown to have a significant protective effect on hypertension^55–59^ and does not adversely affect blood pressure in most people, including those with arterial hypertension^60^.

These results have led to the consideration of antioxidant rich beverages (e.g., coffee) as potentially helpful choices in supporting healthy blood pressures by The European Society of Hypertension (ESH)^61^ and promoting coffee consumption(3–4 cups/day) for the prevention of CVD in the 2021 European Society of Cardiology Guidelines^62^.

However, the mechanisms underlying the protective effects of caffeine remain speculative, and there is a lack of dedicated studies aimed at addressing the impact of different coffee subtypes on clinical outcomes such as CVD, arrhythmia, and mortality.

### The study

The UK Biobank is a large-scale biomedical database and research resource that contains in-depth genetic and health information from over 502,521 UK participants aged between 40 and 69 years^63^. The aim of this study was to provide insights into the impact of coffee on cardiovascular (CV) outcomes, mainly the relationship between coffee consumption and coffee subtypes and new-onset arrhythmias, CVD, and mortality^64^.

Survey and questionnaire responses on lifestyle risk factors and physical examination findings were collected at baseline. Participants were followed up long term to assess health outcomes. Participants with baseline AF, a previous diagnosis of CVD, or missing data regarding coffee intake, coffee type, tea intake, and body mass index (BMI)/smoking/alcohol status were excluded.

Coffee consumption, including type of coffee (instant coffee, ground coffee and/or decaffeinated coffee) and number of cups per day was self-reported on a touchscreen questionnaire. Participants could only select one type of coffee and were grouped into six daily intake categories, consisting of 0, <1, 1, 2–3, 4–5, and >5 cups/day.

Cardiovascular disease was defined as a composite of coronary heart disease (CHD), congestive cardiac failure (CCF), and ischemic stroke. Arrhythmia included ectopy, atrial fibrillation/atrial flutter (AF/ flutter), supraventricular tachycardia (SVT), and ventricular tachycardia (VT)/ventricular fibrillation (VF). Mortality outcomes included all-cause mortality, CV mortality, and sudden cardiac death.

The study adjusted for covariables, including age, gender, alcohol intake, tea intake, obesity, diabetes, hypertension, obstructive sleep apnea, and smoking status. The outcomes of interest were ascertained using the International Classification of Diseases, Tenth Revision (ICD-10) codes available from medical and death records.

## Results of the study

The study included a total of 449,563 UK Biobank participants, free of cardiovascular problems at enrollment (median age 58 years; 55.3% female), of which 100 510 (22.35%) were controls (non-coffee drinkers). The median follow-up time was 12.5 years. The type of coffee, in order of popularity, was instant in 44.1% (*n* = 198,062), ground in 18.4% (*n* = 82,575), and decaffeinated in 15.2% (*n* = 68,416) (Figure 4).

Arrhythmia was diagnosed in 6.7% (*n* = 30,100) participants, which included AF/flutter in 3.4% (*n* = 15,302), SVT in 0.7% (*n* = 3032), and VT/VF in 0.4% (*n* = 2008). Compared to non-drinkers, a U-shaped relationship was observed between increasing levels of coffee consumption and the incidence of any arrhythmia.

The lowest risk for arrhythmias was seen in those who consumed 2–3 coffee cups/day (HR 0.91, CI [0.88–0.94]; *P* < 0.0001). A similar relationship was observed for AF/flutter and SVT (HR 0.88, CI [0.83–0.94], *P* < 0.0001) and VT/VF (HR 0.83, CI [0.70–0.97], *P* = 0.0201) as significant risk reductions were seen in those who consumed 1–5 cups/day, with the lowest risk seen in 4–5 cups/day.

Drinking 1–5 cups/day of ground or instant coffee (but not decaffeinated coffee) was associated with a significant reduction in incident arrhythmia, including AF. The lowest risk was at 4–5 cups/day for ground coffee (HR 0.83; 95% CI [0.76–0.91]; *P* <0.0001) and 2–3 cups/day for instant coffee (HR, 0.88; 95% CI [0.85–0.92]; *P* <0.0001).

Cardiovascular disease was diagnosed in 9.6% (*n* = 43,173) participants during follow-up. A total of 7.7% (*n* = 34,677) participants were diagnosed with incident CHD, 2.8% (*n*= 12,966) with incident CCF, and 1.5% (*n* = 6767) with incident stroke.

Habitual coffee intake of up to 5 cups/day was associated with significant reductions in the risk of incident CVD and CHD (with the lowest risk in those who consumed 2–3 cups/day (HR 0.89, CI [0.86–0.91], *P* < 0.0001), when compared with non-drinkers.

Coffee consumption at all levels was associated with significant reduction in the risk of CCF (lowest risks in those who consumed 2–3 cups/day (HR 0.83, CI [0.79–0.87], *P* < 0.0001) and ischemic stroke (HR 0.84, CI [0.78–0.90], *P* < 0.0001) (Table 1).

A total of 6.2% (*n* = 27,809) participants died during long-term follow-up, including 1.0% (*n* = 4402) from CV causes. A significant reduction in all-cause mortality was associated with coffee consumption up to 5 cups/day, with the greatest effect seen with 2–3 cups/day (HR 0.86, CI [0.83–0.89], *P* < 0.0001).

A significant reduction in CV mortality was observed in coffee drinkers of 1–5 cups/day (lowest risk 1 cup/day; HR 0.82, CI [0.74–0.90], *P* < 0.0001). Coffee intake was not associated with a risk of sudden cardiac death. Consumption of ground coffee at all levels significantly reduced the risk of all-cause and CV mortality.

A sensitivity analysis on coffee drinkers only found no significant difference in the incidence of CVD at all intake categories and across all coffee subtypes. Coffee drinking was associated with a reduction in incident CVD in participants who had normotension (HR 0.90, CI [0.86–0.94], *P* < 0.0001), hypertension (HR 0.91, CI [0.89–0.94], *P* < 0.0001), DM (HR 0.92, CI [0.88–0.97], *P* = 0.001), non-diabetics (HR 0.91, CI [0.88–0.93], *P* < 0.0001), obstructive sleep apnea (OSA) (HR 0.87, CI [0.78–0.96], *P* = 0.008), as well as non-OSA (HR 0.91, CI [0.89–0.94], *P* < 0.0001) when compared with non-drinkers.

## Discussion

This study shows that habitual consumption of all types of coffee reduces the incidence of CVD and all-cause and cardiovascular mortality, with the largest risk reduction of CVD, CHD, CCF, and all-cause mortality seen with the consumption of 2–3 cups of coffee per day. Caffeinated (ground/instant) coffee, but not decaffeinated coffee, was associated with a reduction in new-onset arrhythmias, including AF, with the greatest benefit seen at 4–5 cups/day.

The findings of this study corroborate the beneficial association of habitual coffee intake reported in recent population studies^17,33,34,45,52,65–67^ regarding the risk of stroke^41^ and the reduction in AF incidence^52,53^, CVD/CHD risk^33,34^, and all-cause mortality^40,42,44–47^.

The beneficial effects of coffee on the cardiovascular system might be due to caffeine, which increases endothelial nitric oxide release, downregulates lipogenesis, reduces insulin sensitivity, and has antioxidant properties^48,68,69^.

However, the risk reduction for incidence of CVD, CHD, heart failure, and all-cause and cardiovascular mortality was seen both in the consumption of caffeinated as well as decaffeinated beverages. These results are similar to previous data on the effect of coffee on type-2 DM risk^17^, hypertension^58,59^, all-cause and CV mortality reduction^49–51^.

This finding suggests the importance of the non-caffeinated constituents of coffee, which are likely responsible for the beneficial effects of coffee consumption on CVD and longevity. Interestingly, caffeinated coffee has been associated with a reduction in arrhythmias through unknown mechanisms. Further research is needed to elucidate the protective mechanisms of coffee and the impact of the non-caffeinated constituents of coffee on clinical outcomes.

### Study limitations

The main study limitation is that coffee consumption was self-reported by the study participants, which carries a potential risk of reporting bias. Furthermore, participants could only select one type of coffee in the questionnaire, although some participants might have consumed more than one subtype of coffee over time. Additionally, participants’ coffee consumption was assumed to not change from baseline to follow-up. However, the type of coffee and coffee consumption might have varied throughout the day and over time.

The system used to assess outcomes and track participants’ health (ICD-10 codes) is susceptible to measurement and reporting errors. Furthermore, certain arrhythmias, particularly atrial/ventricular ectopy, may have gone undetected owing to the absence of routine monitoring.

Participants’ alcohol and tea intake were considered in the analysis, but other unaccounted confounding factors, including dietary factors, may have impacted health outcomes. Finally, because the UK Biobank population is predominantly Caucasian, the study conclusions may not be entirely applicable to populations of other ethnicities.

## Lessons learned

The newly published findings indicate that mild-to-moderate consumption of all types of coffee is linked to improved CV outcomes and lower risk of cardiovascular disease and death, with caffeinated coffee significantly reducing the risk of arrhythmias, including AF. Daily coffee intake should not be discouraged by physicians but rather considered part of a healthy lifestyle, even in the presence or newly development of cardiovascular disease, unless there are specific coffee-related personal symptoms.

Whether coffee will be prescribed in the future for the prevention CVD and improvement of cardiovascular health depends on further evaluation of the physiological mechanisms and elucidation of the specific protective effects of coffee consumption.
